# Immune imbalance in the human hippocampus in PTSD revealed by single-nucleus transcriptomics

**DOI:** 10.3389/fimmu.2025.1697171

**Published:** 2026-01-02

**Authors:** Liu Liu, Pengfei Li, Brent A. Wilkerson, Yan Wu, Meng Liu, Roger Shi, Eric D. Hamlett, Steven L. Carroll, Amanda C. LaRue, Zhewu Wang, Hongkuan Fan

**Affiliations:** 1 Department of Pathology and Laboratory Medicine, Medical University of South Carolina, Charleston, SC, United States; 2 Department of Otolaryngology-Head and Neck Surgery, Medical University of South Carolina, Charleston, SC, United States; 3 Department of Psychiatry and Behavioral Sciences, Medical University of South Carolina, Charleston, SC, United States; 4 Academic Magnet High School, North Charleston, SC, United States; 5 Research Service, Ralph H. Johnson Department of Veterans Affairs Health Care System, Charleston, SC, United States

**Keywords:** human hippocampus, immune imbalance, neuroinflammation, PTSD, snRNA sequencing

## Abstract

**Introduction:**

Post-traumatic stress disorder (PTSD) is increasingly recognized as a neuroimmune disorder in which disrupted neuron–glia interactions contribute to long-term cognitive and emotional dysfunction. However, the cellular and molecular basis of immune imbalance in the human hippocampus remains unclear.

**Methods:**

We performed single-nucleus RNA sequencing on postmortem hippocampal tissues from donors with PTSD and matched controls, identifying 10 major cell types, with particular emphasis on neurovascular and glial populations. Differential expression, pathway enrichment, pseudotime trajectory, and cell–cell communication analyses were applied to characterize cellular and molecular alterations.

**Results:**

PTSD samples showed prominent activation of stress-response and inflammatory signaling across astrocytes, microglia, endothelial cells, and mural cells. Microglia and astrocytes underwent robust transcriptional reprogramming with enrichment of immune-related pathways, while endothelial and mural cells exhibited inflammation and impaired blood–brain barrier homeostasis. Trajectory analyses revealed altered state transitions in astrocytes and microglia, indicating dysregulated responses to stress. Furthermore, cell–cell communication analysis uncovered markedly reduced interactions between astrocytes or microglia with excitatory neurons and oligodendrocyte lineage cells, particularly involving stress- and inflammation-related ligand–receptor pairs.

**Discussion:**

These findings demonstrate that PTSD is characterized by immune imbalance at both cellular and intercellular levels, driven by maladaptive glial activation and disrupted neuron–glia communication. Our study provides a comprehensive single-cell atlas of the hippocampal neuroimmune landscape in PTSD and highlights dysfunctional glial–neuronal interactions as a central mechanism underlying disease pathogenesis.

## 1 Introduction

Stress-induced maladaptive responses pose significant public health challenges, affecting a substantial portion of the population (1). Exposure to traumatic events—whether acute or chronic, single or repeated—can lead to the development of post-traumatic stress disorder (PTSD) in humans and PTSD-like phenotypes in animal models (1–3). PTSD, a stressor- and trauma-related mental disorder, has a global lifetime prevalence of approximately 5.6% (4) and imposes a considerable burden on individuals and society (4, 5). Among U.S. veterans with PTSD, the prevalence of chronic pain has been reported to be as high as 66% (6). Furthermore, PTSD has been associated with an increased risk of chronic conditions, including cardiovascular and autoimmune diseases, as well as accelerated biological aging and elevated premature mortality (4, 7, 8). Despite these serious outcomes, the underlying mechanisms driving PTSD pathophysiology remain poorly understood.

Emerging evidence suggests that stress-related immune dysregulation within the central nervous system (CNS) plays a critical role in the pathogenesis of PTSD (9). Preclinical models of PTSD have demonstrated that neuroinflammation is closely associated with both the development and persistence of PTSD-like phenotypes (8, 10). Notably, increased levels of pro-inflammatory cytokines in the hippocampus have been observed in animal models, correlating with behavioral manifestations characteristic of PTSD (9, 11, 12). In clinical studies, significant reductions in hippocampal volume have also been reported in individuals with PTSD compared to controls (8). Thus, elucidating the mechanisms of hippocampal neuroinflammation in PTSD may offer critical insights into novel strategies for prevention and treatment.

Neuroinflammation is orchestrated by resident immune cells, including microglia and astrocytes, as well as infiltrating peripheral mononuclear cells—a process facilitated by increased permeability of the blood–brain barrier (8, 13). Among CNS-resident immune cells, microglia are a major source of pro-inflammatory cytokines and act as first responders to injury and stress-related stimuli (9, 14, 15). Rodent models have demonstrated that psychological stress induces a pro-inflammatory phenotype in microglia (8, 16). Notably, stress-induced microglial activation in the hippocampus may represent a key mechanistic link underlying the comorbidity between PTSD and chronic pain (9). In parallel, astrocytes also play a significant role in neuroinflammatory processes across various neurological conditions, including those associated with injury and trauma (8). Specifically, hippocampal astrocytes have been identified as a primary source of interleukin-1β (IL-1β) in response to stress (17, 18). Despite growing evidence implicating glial cells in the pathophysiology of PTSD, the distinct roles and interactions among immune cell populations in the hippocampus remain poorly defined.

Recent single-cell and spatial transcriptomic studies of postmortem prefrontal cortex tissue from individuals with PTSD have begun to map gene expression changes and disrupted intercellular communication networks associated with the disorder (19, 20). These studies have identified PTSD-related transcriptional alterations in microglia, astrocytes, endothelial cells, and neurons. However, comparable analyses of human hippocampal tissue—a region critically implicated in PTSD—remain limited.

In this study, we applied single-nucleus RNA sequencing (snRNA-seq) to postmortem hippocampal tissue from individuals with PTSD to characterize cellular and molecular alterations within immune-related compartments. We examined dynamic changes in cell states and intercellular communication, uncovering a central role for glial–neuronal interactions in mediating immune dysregulation in PTSD. These findings provide novel insights into the pathophysiology of PTSD and highlight potential targets for therapeutic intervention.

## 2 Result

### 2.1 Single-nucleus RNA sequencing identifies major cell populations in the human hippocampus from control and PTSD samples

**Figure 1:**
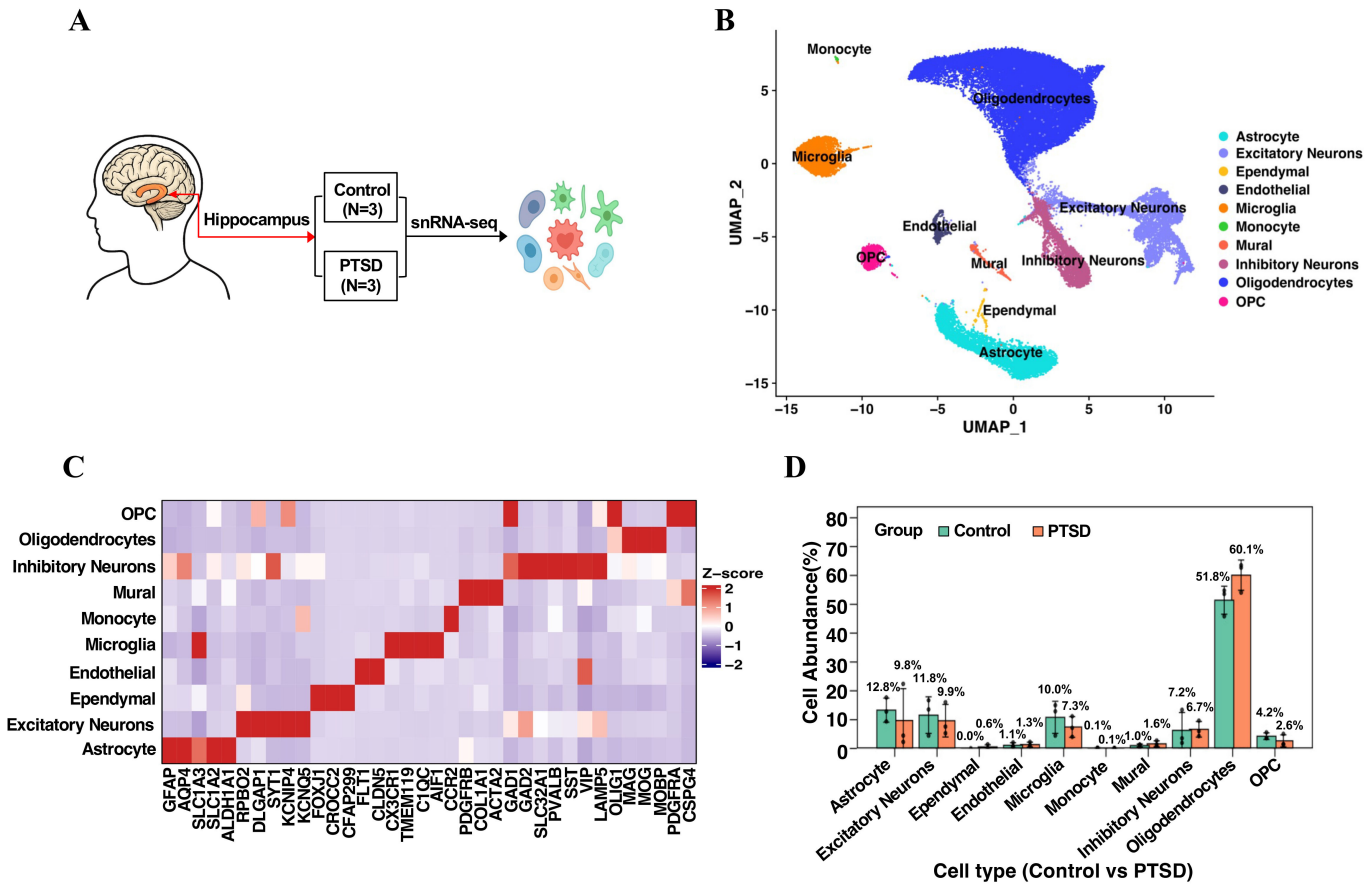
Single-nucleus transcriptomic profiling of the human hippocampus. **(A)** Schematic workflow for nuclei isolation, library preparation, sequencing and computational analysis. **(B)** UMAP projection of integrated snRNA-seq data showing 10 transcriptionally distinct clusters annotated by canonical marker genes. **(C)** Heatmap of scaled expression (Z-score) for canonical marker genes used for cluster annotation. Boxes highlight genes used to identify astrocytes and microglia. **(D)** Bar plot showing the relative abundance (%) of each cell type in control and PTSD groups. Individual data points for each sample are displayed to illustrate variability, and bars represent the mean ± standard error.

To characterize cell type–specific transcriptomic alterations in the hippocampus of PTSD patients, we performed single-nucleus RNA sequencing (snRNA-seq) on postmortem hippocampal tissues from six donors, including three controls and three PTSD cases (**Figure 1A**). After quality control and integration, high-quality nuclei were obtained and visualized using uniform manifold approximation and projection (UMAP), which resolved into 10 transcriptionally distinct clusters corresponding to major neural and non-neural cell types, including astrocytes, excitatory neurons, inhibitory neurons, oligodendrocytes, oligodendrocyte precursor cells (OPCs), endothelial cells, mural cells, microglia, monocytes, and ependymal cells (**Figure 1B**). Cell type identity was assigned based on the expression of canonical marker genes (**Figure 1C**). To further explore donor-specific patterns, UMAP visualizations were generated for each of the three control and three PTSD samples, confirming consistent clustering of the 10 cell types across all donors (**Supplementary Figure S1A**). Quantitative analysis of donor-level cell proportions revealed group-specific shifts in glial immune populations. As a proportion of the total cell nuclei, astrocytes were reduced by 23% in PTSD relative to controls (9.8% vs. 12.8%), while microglia exhibited a 27% decrease (7.3% vs. 10%) (**Figure 1D**). These cell proportion differences were further validated by comparing the relative abundance of the 10 cell types across the three control and three PTSD samples, which showed consistent trends in glial population shifts (**Supplementary Figure S1B**). Although some changes are numerically small, the consistent directionality across donors motivated focused downstream analyses of transcriptional programs in astrocytes and microglia. These results establish a cellular atlas of the human hippocampus in PTSD and highlight cell type composition changes that may contribute to disease-related neuropathology.

### 2.2 Cell type–specific transcriptional alterations in stress response and inflammation pathways in PTSD hippocampus

**Figure 2:**
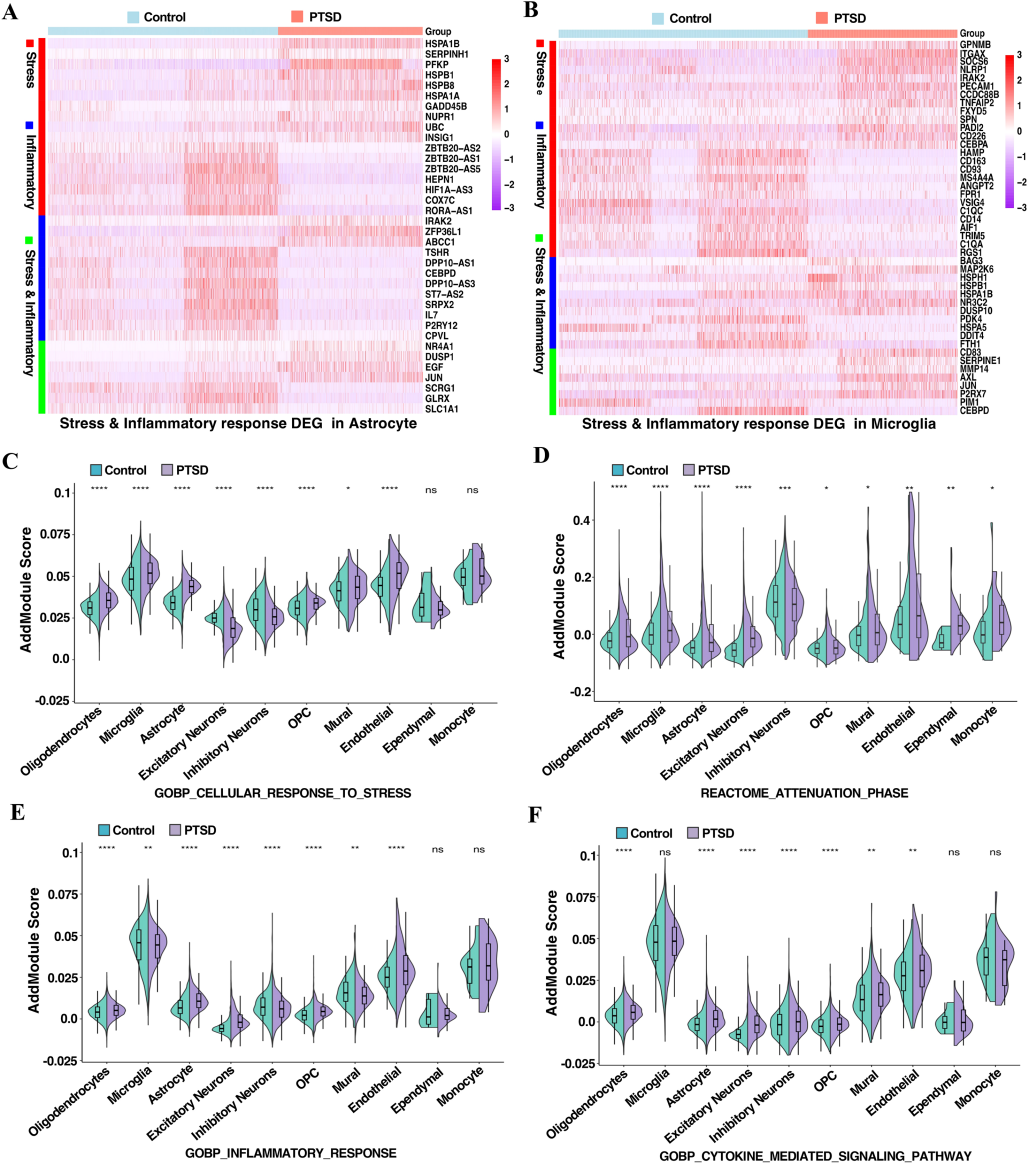
Cell type–specific differential gene expression and pathway activity changes in PTSD versus control hippocampus. **(A)** Heatmap showing differentially expressed genes (DEGs) in astrocytes. Red, stress response–related genes; blue, inflammation-related genes; green, genes involved in both stress response and inflammation. Expression values represent scaled average expression across cells, highlighting coordinated upregulation of stress- and immune-related programs in PTSD compared to control. **(B)** Heatmap showing DEGs in **microglia** categorized into the same three functional modules: Red, stress response–related genes; blue, inflammation-related genes; green, genes involved in both stress response and inflammation **(C–F)** Module scoring of selected gene sets across 11 hippocampal cell types in PTSD versus controls. **(C)** Gobp_cellular_response_to_stress: PTSD samples showed significantly higher scores in oligodendrocytes, microglia, astrocytes, OPCs, mural cells, and endothelial cells, with reductions in excitatory and inhibitory neurons. **(D)** Reactome_attenuation_phase: PTSD samples exhibited elevated scores in oligodendrocytes, microglia, astrocytes, excitatory neurons, and several non-neuronal cell types, but reduced scores in inhibitory neurons and endothelial cells. **(E)** Gobp_inflammatory_response: PTSD was associated with increased inflammatory response scores in astrocytes, oligodendrocytes, endothelial cells, and excitatory neurons, but decreased scores in microglia, inhibitory neurons, and mural cells. **(F)** Gobp_cytokine_mediated_signaling_pathway: Astrocytes, oligodendrocytes, excitatory neurons, OPCs, mural cells, and endothelial cells displayed higher scores in PTSD, whereas inhibitory neurons were reduced, and microglia and monocytes showed no significant differences. ****p < 0.0001, ***p < 0.001, **p < 0.01, *p < 0.05, ns = not significant.

To explore cell type–specific transcriptional alterations in PTSD hippocampus, we performed differential expression and pathway enrichment analyses with a focus on astrocytes and microglia. In astrocytes, we identified a marked upregulation of stress response–related genes, including *PFKP*, *HSPA1B*, *HSPA1A* (21), and *HSPB1* (22), along with downregulation of *HEPN1*, *ZBTB20-AS5*, and *RORA-AS1*. Inflammatory genes such as *ZFP36L1* (23), *IRAK2*, and *ABCC1* were significantly upregulated, while *DPP10-AS3*, *DPP10-AS1*, and *IL7* were downregulated. Notably, dual-function genes implicated in both stress and inflammation (*EGF* (24), *DUSP1* (25), *JUN* (26)) were upregulated, suggesting a coordinated activation of stress-adaptive and immune pathways (**Figures 2A**, **S2A**). In microglia, PTSD was associated with increased expression of stress-related genes (*ITGAX*, *NLRP1*, *SOCS6*, *PECAM1*) and inflammation-associated genes (*MAP2K6*, *NR3C2*, *IRAK2*), along with upregulation of dual-function mediators (*P2RX7* (27), *CD83*, *JUN*, *SERPINE1*). Conversely, several homeostatic microglial markers (*HAMP*, *CD163*, *C1QC*, *MS4A4A*, *FTH1*) were downregulated, indicating a shift toward a reactive state (28) (**Figures 2B**, **S2B**). To further characterize neuronal and glial alterations, differential expression analysis was extended to excitatory neurons, inhibitory neurons, and oligodendrocytes. In excitatory neurons, the differentially expressed genes included a mix of upregulated stress- and inflammation-related genes and downregulated synaptic function genes (**Supplementary Figure S2C**). Similarly, inhibitory neurons showed significant differential expression genes of stress response and inflammatory response (**Supplementary Figure S2D**). In oligodendrocytes, the differentially expressed genes highlighted alterations in stress response-related genes and inflammatory response pathways (**Supplementary Figure S2E**). To experimentally validate the transcriptional alterations identified in our snRNA-seq analysis, we performed quantitative PCR (qPCR) using total RNA extracted from post-mortem hippocampal tissue of Control and PTSD individuals. We selected representative DEGs from astrocyte-, microglia-, and neuron-associated gene modules defined by our snRNA-seq analysis. Consistent with the directionality observed in the single-nucleus dataset, qPCR confirmed significant upregulation of *HSPA1A*, *HSPB1*, and *DUSP1*, and downregulation of *ZBTB20* and *IL7*, which were originally identified within astrocyte-associated DEG clusters. Similarly, qPCR validated the increased expression of *ITGAX*, *NLRP1*, *SOCS6*, *PECAM1*, and *MAP2K6*, and the reduced expression of *CD163* and *C1QC*, which were identified in microglia-associated DEG modules. Finally, *HSPA1A* and *ENPP2*, which we identified within neurons/oligodendrocyte-associated DEGs, also showed significant upregulation in bulk tissue. These results (**Supplementary Figure S2F**) provide independent validation of the overall expression trends observed in the snRNA-seq analysis, supporting the robustness of our computational findings. Module scoring of predefined gene sets further revealed cell type–specific differences (19). The stress response pathway (Gobp_cellular_response_to_stress) was significantly enriched in astrocytes, microglia, and several glial subtypes in PTSD (19) (**Figure 2C**), whereas both excitatory and inhibitory neurons exhibited reduced activity. The attenuation phase pathway (Reactome_attenuation_phase), which restore cellular homeostasis and prevent overactivation of stress pathways, showed broad activation across glia, excitatory neurons and endothelial but was suppressed in inhibitory neurons (**Figure 2D**). The inflammatory response (Go_inflammatory_response) was elevated in astrocytes but unexpectedly reduced in microglia (29), suggesting a possible cell type–specific immune imbalance (**Figure 2E**). Cytokine-mediated signaling (Gobp_cytokine_mediated_signaling_pathway) was enhanced in astrocytes and multiple non-neuronal cell types, with no significant change in microglia (**Figure 2F**). Together, these results indicate that PTSD hippocampus is characterized by a pronounced activation of stress and inflammatory pathways in astrocytes, accompanied by altered immune signatures and potential functional reprogramming in microglia, pointing to a dysregulated glia-mediated immune environment.

### 2.3 Stress-response–dominated transcriptional programs in astrocytes and microglia reflect immune imbalance

**Figure 3:**
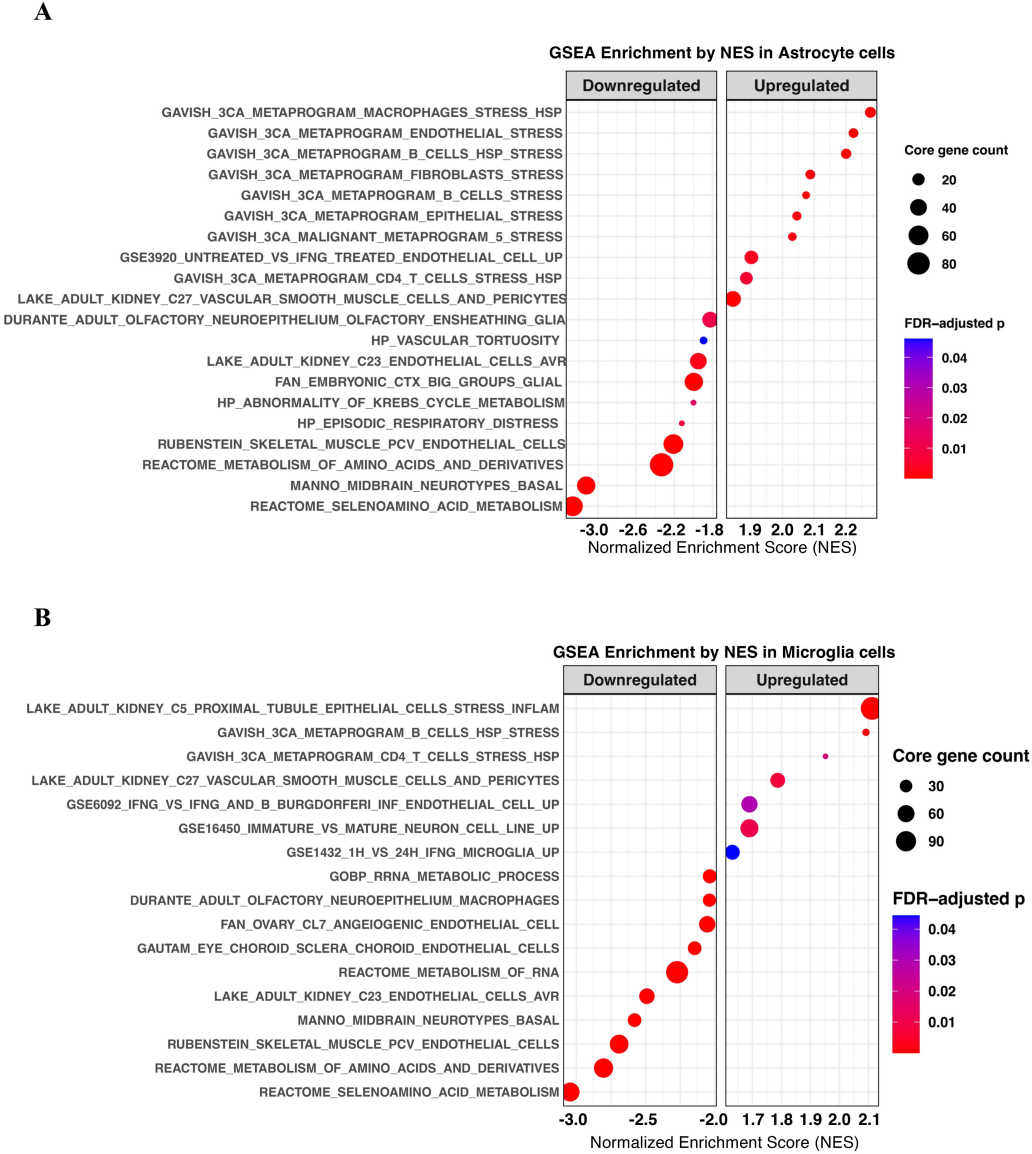
Gene set enrichment analysis (GSEA) reveals stress-response–oriented transcriptional programs in astrocytes and microglia of the PTSD hippocampus. **(A)** GSEA of astrocytes identified the top 20 activated and suppressed pathways. Prominent enriched signatures included stress-related programs such as *Gavish_3CA_metaprogram_macrophages_ stress_hsp*, *Gavish_3CA_metaprogram_endothelial_stress*, and *Gavish_3CA_metaprogram_B_ cells_ hsp_stress*. **(B)** GSEA of microglia revealed significant enrichment of stress-response–associated pathways, including *Gavish_3CA_ metaprogram_B_cells_hsp_stress*, and *Gavish_3CA_metaprogram_cd8_T_cells_stress_hsp*. Together, these findings highlight a stress-adaptive rather than pro-inflammatory transcriptional state of astrocytes and microglia in the PTSD hippocampus.

Building on the immune imbalance observed in **Figures 1** and **2**, we next examined whether astrocytes and microglia—two critical immune-responsive cell types in the hippocampal neurovascular unit—exhibited coordinated transcriptional alterations in PTSD. To this end, we performed Gene Set Enrichment Analysis (GSEA) separately in astrocytes and microglia (**Figure 3**). In astrocytes, the enriched pathways were dominated by cellular stress-response programs rather than classical immune activation. Among the top 20 activated or suppressed gene sets, several were significantly associated with stress-related signatures, including *Gavish_3CA_metaprogram_macrophages_stress_hsp*, *Gavish_3CA_metaprogram_endothelial_stress*, and *Gavish_3CA_ metaprogram _B_cells_hsp_stress* (**Figure 3A**). A similar pattern was observed in microglia, which also showed significant enrichment of stress-related programs, such as *Gavish_3CA_metaprogram_B_cells_hsp_stress*, and *Gavish_3CA_metaprogram_cd4_T_cells _stress_hsp* (30) (**Figure 3B**). To further investigate whether this stress-centered transcriptional profile extended to other cell types, we conducted GSEA in excitatory neurons, inhibitory neurons, and oligodendrocytes. In excitatory neurons, GSEA revealed significant enrichment of upregulated stress response gene sets, including those related to heat shock and cellular stress adaptation (**Supplementary Figure S3A**). Similarly, inhibitory neurons exhibited significant enrichment of upregulated stress response gene sets, consistent with the patterns observed in glial cells (**Supplementary Figure S3B**). In oligodendrocytes, GSEA also identified significant enrichment of upregulated stress response gene sets, indicating a broad involvement of stress-adaptive programs across multiple cell types (**Supplementary Figure S4**). Rather than mounting classical inflammatory programs, both astrocytes and microglia in PTSD hippocampus appear to preferentially engage stress-adaptive transcriptional programs. This shift suggests that the immune imbalance in PTSD is not simply a matter of heightened or suppressed inflammation, but rather a redirection of glial immune responsiveness toward cellular stress adaptation. Collectively, these findings indicate that the hippocampal immune microenvironment in PTSD is characterized by a stress-centered, non-canonical form of immune imbalance.

### 2.4 Astrocyte trajectory analysis reveals altered cellular states in PTSD hippocampus

**Figure 4:**
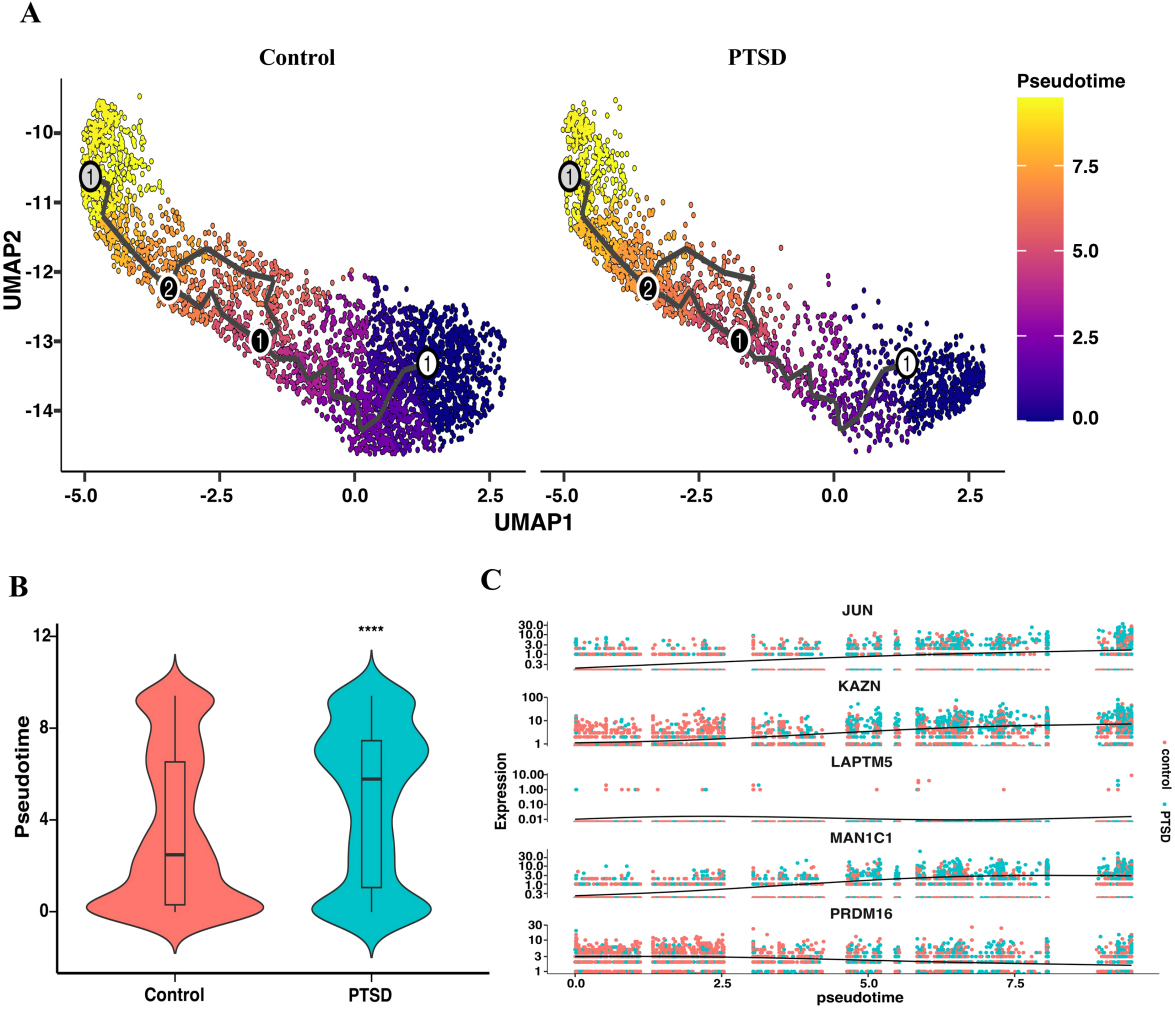
Pseudotime trajectory analysis of astrocytes in control and PTSD hippocampus. **(A)** UMAP visualization of astrocyte trajectories in control and PTSD samples, colored by pseudotime progression. **(B)** Violin plots depicting the distribution of pseudotime values between control and PTSD groups, showing a significant shift toward higher pseudotime values in PTSD astrocytes. **(C)** Representative genes dynamically regulated along pseudotime, including *JUN*, *KAZN*, *LAPTM5*, *MAN1C1*, and *PRDM16*, highlighting transcriptional reprogramming associated with altered astrocyte states in PTSD.

Having established that astrocytes and microglia preferentially engage stress-related transcriptional programs rather than inflammatory pathways in PTSD hippocampus (**Figures 2**, **3**), we next asked whether astrocytes also exhibit altered state transitions consistent with immune imbalance. To address this, we performed pseudotime trajectory analysis of astrocytes (**Figure 4**). Trajectory reconstruction revealed a broadly conserved astrocytic developmental path across control and PTSD samples (**Figure 4A**). However, quantitative comparison showed that PTSD astrocytes were significantly shifted toward later pseudotime states relative to controls (**Figure 4B**), suggesting a bias toward more “activated” or stress-adaptive cellular states. To further characterize this trajectory-associated reprogramming, we identified the top genes whose expression varied along pseudotime. These included *JUN*, *KAZN*, *LAPTM5*, *MAN1C1*, and *PRDM16* (**Figure 4C**), genes implicated in stress signaling, immune modulation, and cellular plasticity (31). Together, these results demonstrate that PTSD astrocytes not only adopt stress-response transcriptional signatures but also undergo altered state transitions along pseudotime. This dynamic shift reinforces the notion that immune imbalance in the PTSD hippocampus is characterized by stress-driven reprogramming of glial states rather than canonical inflammatory activation.

### 2.5 Microglia trajectory analysis reveals altered cellular dynamics and transcriptional programs in PTSD

**Figure 5:**
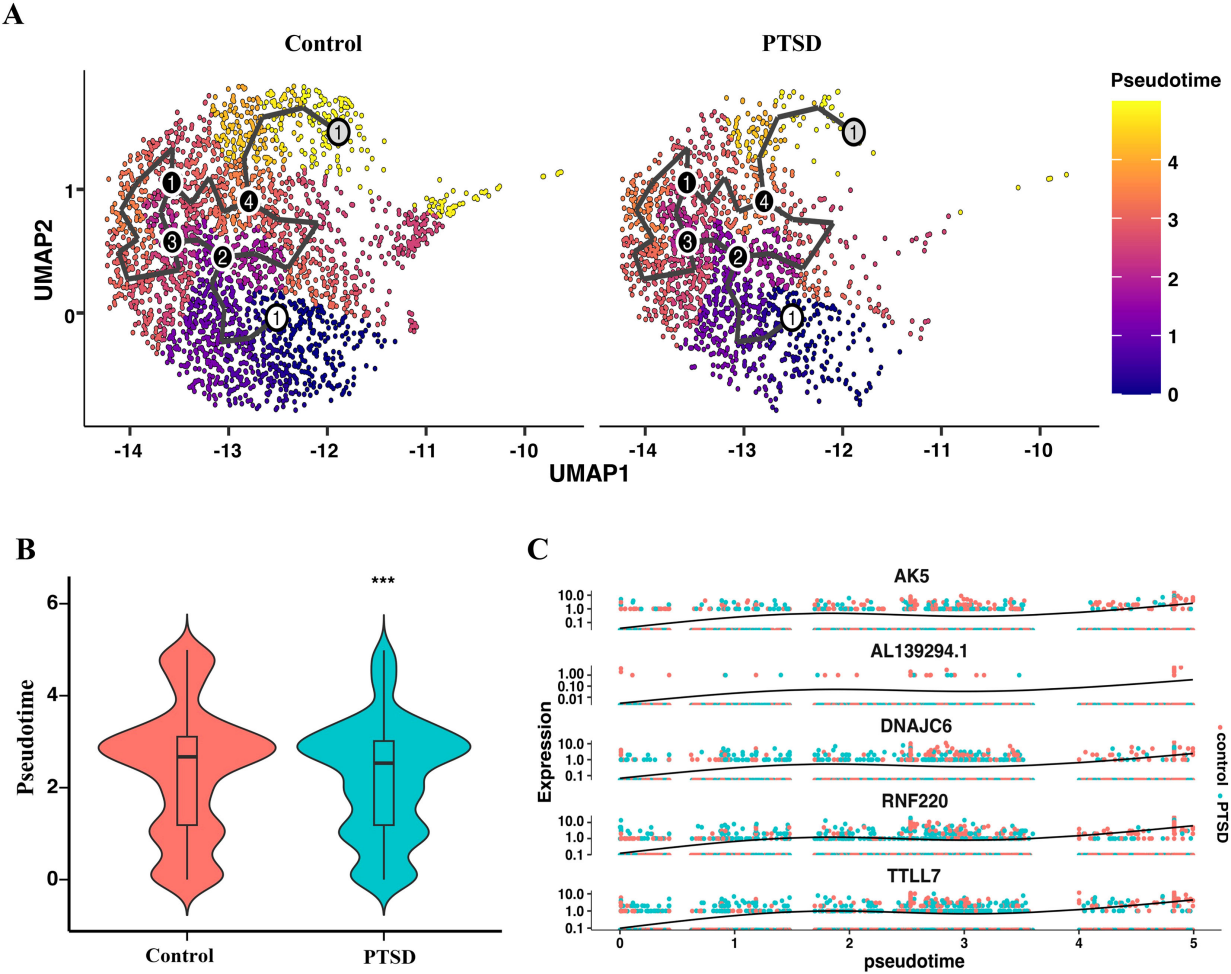
Pseudotime trajectory analysis of Microglia in Control and PTSD hippocampus. **(A)** UMAP embedding of Microglia cells from Control and PTSD groups, with pseudotime trajectory inferred by Monocle3. Cells are colored by pseudotime progression. **(B)** Quantification of pseudotime distributions between groups, shown as violin plots. Microglia from PTSD samples display significantly lower pseudotime values compared to Controls, suggesting an altered cellular state transition pattern. **(C)** Top 5 genes (AK5, AL139294.1, DNAJC6, RNF220, TTLL7) dynamically regulated along pseudotime trajectory were identified, highlighting transcriptional programs associated with impaired microglial adaptation in PTSD.

To further dissect the cellular dynamics underlying immune imbalance in PTSD, we performed pseudotime trajectory analysis of microglia (**Figure 5A**), which allow us to capture the continuum of microglial activation and functional state transitions. Compared with control, microglia in PTSD samples exhibited a left-shifted pseudotime distribution, as quantified by violin plot analysis (**Figure 5B**). This indicates that PTSD-associated microglia are enriched in earlier pseudotime states, in contrast to astrocytes which showed relatively advanced trajectories, reflecting cell-type–specific dysregulation of immune adaptation. Moreover, trajectory-based gene expression analysis revealed key transcripts dynamically regulated along pseudotime, including *AK5*, *AL139294.1*, *DNAJC6*, *RNF220*, *and TTLL7* (**Figure 5C**). These genes are implicated in neuronal signaling, protein modification, and stress response, suggesting that impaired microglia progression may compromise their immune-modulatory and neuroprotective functions (32). Together, these findings highlight that PTSD disrupts the temporal dynamics of microglia, contributing to the broader pattern of immune imbalance across hippocampal cell types.

### 2.6 PTSD disrupts astrocyte– and microglia–neuron communication, particularly through stress- and inflammation-related ligand–receptor interactions

**Figure 6:**
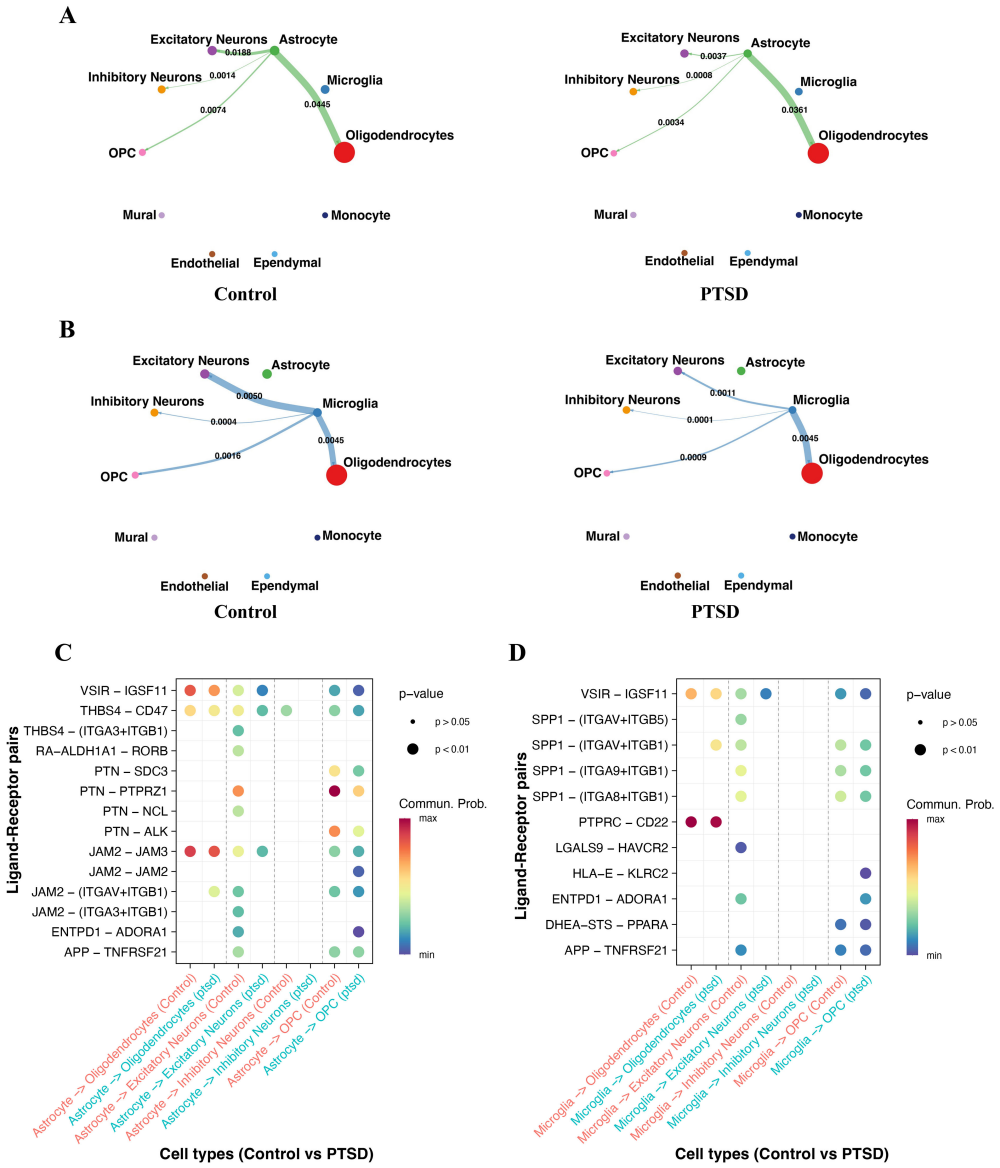
Cell–cell communication analysis reveals weakened astrocyte– and microglia–neuron interactions in PTSD. **(A)** CellChat analysis of astrocyte interactions with excitatory neurons, inhibitory neurons, oligodendrocyte precursor cells (OPCs), and oligodendrocytes between Control and PTSD groups. Astrocyte–excitatory neuron and astrocyte–OPC communication was significantly reduced in PTSD. **(B)** CellChat analysis of microglia interactions with excitatory neurons, inhibitory neurons, OPCs, and oligodendrocytes. Microglia–excitatory neuron and microglia–OPC communication were markedly decreased in PTSD. **(C)** Ligand–receptor pair analysis of stress response and inflammation pathways in astrocyte communication. Astrocyte–excitatory neuron interactions in these pathways were significantly reduced in PTSD, whereas changes with other cell types were minimal. **(D)** Ligand–receptor pair analysis of stress response and inflammation pathways in microglia communication. Similarly, microglia–excitatory neuron interactions in stress- and inflammation-related signaling were substantially reduced in PTSD, with limited alterations in other connections.

To further explore how neuroimmune imbalance in PTSD impacts intercellular crosstalk, we performed cell–cell communication analysis focusing on astrocyte– and microglia–mediated signaling. We observed that both astrocytes and microglia exhibited weakened communication with excitatory neurons and OPCs in PTSD compared with controls, indicating a loss of critical neuro–glial support (**Figures 6A, B**). Importantly, when restricting the analysis to stress response and inflammation-related ligand–receptor interactions, astrocyte– and microglia–excitatory neuron connections showed the most pronounced reduction in PTSD, while their communication with inhibitory neurons and oligodendrocytes remained relatively stable (**Figures 6C, D**). These findings highlight that PTSD-associated immune imbalance not only alters intrinsic cellular states but also disrupts neuron–glia signaling networks, particularly stress- and inflammation-driven interactions, which may underlie synaptic vulnerability and impaired circuit resilience in the disorder.

## 3 Discussion

In this study, we performed single-cell RNA sequencing of hippocampal tissues from individuals with post-traumatic stress disorder (PTSD) and controls to delineate transcriptional and cellular alterations within the neurovascular unit (NVU) and neuronal compartments. Our findings provide new insights into how immune imbalance and impaired cell–cell communication converge to disrupt hippocampal homeostasis in PTSD (32).

First, unbiased clustering revealed significant transcriptional heterogeneity across hippocampal cell types. Notably, astrocytes, microglia—core components of the NVU—displayed profound alterations in PTSD. Differential expression and pathway enrichment analyses consistently indicated that these NVU-related populations exhibited upregulation of stress response and immune-related pathways (30). These results suggest that PTSD is associated with a dysfunctional NVU, which may compromise vascular stability and impair neuronal support. Second, astrocytes and microglia, as key immune-responsive glial cells, exhibited distinct transcriptional signatures. In PTSD, both cell types showed reduced activation of stress response pathways and diminished expression of pro-inflammatory signaling molecules, indicating a state of “immune imbalance.” Rather than reflecting hyper-inflammation, our results point toward an aberrant suppression or dysregulation of immune function, which may weaken the brain’s resilience to stress exposure (33). Microglia in particular showed attenuated expression of immune effector genes while astrocytes displayed reduced capacity for neurotrophic support, suggesting that impaired glia-mediated immune surveillance may contribute to long-term vulnerability in PTSD. Importantly, our analysis revealed robust upregulation of stress-inducible molecular chaperones and immediate early genes, including members of the HSP family (*HSPA1A*, *HSPA1B*, *HSP90AA1*, *DNAJB1*) and the phosphatase *DUSP1 (*34). These transcriptional changes reflect activation of cellular stress-response programs in astrocytes and microglia, rather than differential expression of canonical GR pathway components (e.g., *NR3C1*, *FKBP5*, *TSC22D3*, *SGK1*) (20, 35). Notably, previous PTSD studies have consistently reported dysregulation of the hypothalamic–pituitary–adrenal (HPA) axis and impaired glucocorticoid receptor (GR) signaling (36–38). Our findings suggest that, although direct GR target genes were not significantly altered in our dataset, stress-responsive programs mediated by heat shock proteins and DUSP1 may represent downstream consequences of HPA axis dysfunction and contribute to maladaptive neurovascular unit (NVU) responses in PTSD.

Moreover, the glial alterations we observed—particularly astrocyte reduction and microglial state shifts—likely have direct implications for hallmark PTSD symptoms such as impaired fear extinction, memory dysregulation, and heightened arousal. For instance, hippocampal astrocyte depletion and morphological remodeling have been associated with maladaptive memory consolidation and fear retention in stress-related disorders, indicating their role in sustaining traumatic memory circuits (39). Moreover, *in vivo* imaging studies using TSPO tracers revealed reduced microglial activation in prefrontal–limbic regions of individuals with PTSD, with lower TSPO availability correlating with greater symptom severity, particularly anhedonia and emotional dysregulation (29). These observations suggest that the immune imbalance we characterize—marked by suppressed glial responsiveness and disrupted neuron–glia communication—may contribute to key PTSD phenotypes, such as impaired contextualization of traumatic memories and reduced hippocampal plasticity. Third, trajectory analysis further highlighted altered cell state dynamics. Astrocytes and microglia in PTSD displayed distinct pseudotime trajectories, consistent with transcriptional reprogramming under chronic stress conditions. These findings imply that PTSD-related glial dysfunction is not merely a static alteration but represents a dynamic shift in cellular state, which may underlie persistent neurobiological changes after trauma (31).

Importantly, our cell–cell communication analysis provided mechanistic insights into how immune imbalance may propagate across the hippocampal microenvironment. We observed marked reductions in communication strength between astrocytes or microglia and excitatory neurons as well as oligodendrocyte precursor cells (OPCs) in PTSD. Specifically, signaling mediated by stress response- and inflammation-related ligand–receptor pairs was significantly weakened in PTSD, particularly in astrocyte–neuron and microglia–neuron interactions (40). These findings suggest that disrupted glia–neuron communication may impair synaptic regulation and neuroplasticity, ultimately contributing to hippocampal dysfunction and the cognitive and emotional symptoms of PTS. Taken together, our results support a model in which PTSD is characterized by (i) NVU dysfunction, (ii) glial immune imbalance, and (iii) impaired glia–neuron communication. Rather than an overt pro-inflammatory state, PTSD appears to involve maladaptive immune suppression or dysregulation, which compromises the brain’s capacity to respond to stress and maintain homeostasis. The observed reduction in astrocyte- and microglia-mediated signaling to neurons and OPCs may explain persistent impairments in synaptic remodeling and myelination that are critical for cognitive resilience.

These findings extend previous reports linking PTSD to neuroinflammation and vascular dysfunction, and they highlight the NVU as a central target of stress-induced pathology (41). Importantly, our results argue for a nuanced view of immune dysregulation in PTSD: not simply hyperactivation, but rather an imbalance characterized by impaired stress responsiveness and weakened neuro-immune crosstalk. From a translational perspective, strategies aimed at restoring NVU integrity, normalizing glial function, and enhancing glia–neuron communication may hold therapeutic potential for PTSD. For example, pharmacological modulation of astrocytic or microglial signaling pathways, or interventions targeting BBB stability, could represent promising avenues for future research.

In this study, we provide a new and important contribution by generating and analyzing single-cell RNA sequencing data from the human hippocampus of patients with PTSD and control individuals. To our knowledge, this represents the first single-cell transcriptomic study of the human hippocampus in PTSD, systematically dissecting neurovascular and glial populations at single-cell resolution and offering unprecedented insights into cellular and molecular alterations associated with PTSD-induced neurovascular dysfunction. Several limitations should be noted. First, the sample size was modest, reflecting the challenges of obtaining high-quality postmortem hippocampal tissue. Second, while single-cell RNA sequencing provides rich transcriptional data, it does not directly capture protein-level or functional changes. Future studies integrating spatial transcriptomics, proteomics, and *in vivo* functional assays will be critical to validate and extend our findings. Finally, it remains unclear whether the observed immune imbalance is a cause or consequence of PTSD; longitudinal and experimental models will be necessary to establish causal relationships. It is important to emphasize that, while our single-nucleus transcriptomic analyses identify disrupted glial–neuronal interactions as a prominent feature of PTSD, the present study cannot determine whether these alterations actively drive disease development or represent downstream consequences of chronic stress exposure. Establishing a causal role for these cell–cell communication deficits will require targeted experimental perturbations—such as cell type–specific genetic knockdown, CRISPR-based modulation, or pharmacological inhibition of key ligand–receptor pathways—in animal models or human-derived cellular systems. Such mechanistic studies are beyond the scope of the current work but will be essential to validate whether restoring glia–neuron communication can ameliorate PTSD-related phenotypes. Supporting our findings, mouse models of PTSD have shown that microglial and astrocyte activation contribute to PTSD-like behaviors (42, 43).

In conclusion, our single-nuclear transcriptomic analysis reveals that PTSD is associated with profound alterations in the hippocampal NVU, characterized by immune imbalance and disrupted cell–cell communication. These findings advance our mechanistic understanding of PTSD pathophysiology and underscore the potential of targeting neurovascular and glial pathways to restore hippocampal function and resilience.

## 4 Materials and methods

### 4.1 Human hippocampal tissue collection

Postmortem human hippocampal brain tissues were obtained from the NIH NeuroBioBank and the Medical University of South Carolina (MUSC) Brain Bank. Samples were collected from six individuals: three diagnosed with PTSD and three age-matched control subjects. For validation, we analyzed four postmortem hippocampal tissue samples from subjects with PTSD along with four corresponding control samples.

### 4.2 Single-nucleus RNA sequencing and initial data processing

Frozen tissue samples were shipped to Novogene (San Jose, CA), where nuclei were isolated by homogenizing the tissue, filtering the homogenate to remove debris, and pelleting the nuclei by centrifugation. Single-cell 3′ RNA libraries were prepared using the 10x Genomics Chromium Single Cell 3′ v3.1 platform. Libraries were sequenced on an Illumina NovaSeq X Plus system with a target depth of approximately 50,000 read pairs per nucleus. Initial data processing was performed by Novogene, including alignment to the GRCh38 human reference genome. Raw sequencing data were further processed using Cell Ranger (10x Genomics) with default parameters, including unique molecular identifier (UMI) collapsing, barcode filtering, and generation of gene expression matrices.

### 4.3 Filtering, normalization, integration, and clustering

Subsequently, batch correction and downstream analyses were performed using the Seurat v5.0.1. Cells with fewer than 200 detected genes, more than 5,000 genes, or over 10% mitochondrial gene content were filtered out to remove low-quality or dying cells. Genes expressed in fewer than 3 cells were also excluded. Hippocampal snRNA-seq samples were normalized using SCTransform (vst.flavor = “v2”, return.only.var.genes = FALSE) with regression of sequencing depth, mitochondrial transcript percentage, and complexity and retaining all genes. Shared genes were identified (SelectIntegrationFeatures) and integration features were centered and scaled (PrepSCTIntegration). Highly variable genes were identified and used for dimensionality reduction via principal component analysis (PCA) and integration anchors were identified by FindIntegrationAnchors (reduction = “rpca”, dims = 1:50, k.anchor = 20. Samples were integrated using IntegrateData (normalization.method = “SCT”, dims = 1:50, k.weight = 100).

PCA was run again on the integrated dataset using RunPCA (npcs = 50), the SNN graph was constructed using FindNeighbors, and UMAP was constructed using PC. Clusters were identified by FindClusters (algorithm = 1, n.iter = 100) at a range of resolutions; resolution of 0.7 was selected based on cluster stability in clustree plots. The resulting clusters represent transcriptionally distinct subpopulations showing enrichment in established cell-specific markers. The data were then normalized using the “LogNormalize” method with a scaling factor of 10,000.

Quality control metrics, including the number of detected genes per cell, UMI counts, and mitochondrial gene percentages, were visualized using Dimplot in each sample to confirm the removal of low-quality cells and correction for technical covariates such as depth and complexity.

### 4.4 Marker gene identification and cell type annotation

To identify marker genes for each cluster, differential gene expression analysis was performed using the Wilcoxon rank-sum test implemented in the FindAllMarkers function of the Seurat package. Genes with an adjusted p-value (q-value) < 0.05 and log_2_ fold change > 0.25 were considered significantly differentially expressed. Cluster-specific marker genes were then compared against known canonical markers to assign cell-type identities to each cluster.

### 4.5 Differential expression and gene set enrichment analysis

Differentially expressed genes (DEGs) between PTSD and control groups were identified within specific cell types using Seurat’s FindMarkers() function (min.pct = 0.1 and logfc.threshold = 0), meaning that all genes expressed in at least 10% of cells were tested regardless of fold change. Genes with adjusted p < 0.05 and |log2 fold change| > 1 were considered significant. Gene Set Enrichment Analysis (GSEA) was conducted using the fgsea R package against MSigDB Hallmark gene sets and curated gene ontology (GO) terms related to inflammation and blood–brain barrier (BBB) function. To ensure an unbiased and comprehensive pathway screening, we used curated gene sets from MSigDB (including Hallmark, GO, Reactome, and select published gene sets such as Gavish et al.), as many of these signatures represent broadly conserved cellular programs (e.g., stress response, metabolic regulation, transcriptional control) relevant beyond their original disease context.

### 4.6 Pseudotime trajectory analysis

To investigate dynamic transcriptional changes of specific cell type during PTSD, we performed pseudotime trajectory analysis using Monocle 3 (v1.3.7). We focused on specific cell types of interest, which were subset from a pre-processed and integrated Seurat object that had undergone SCTransform normalization, clustering, and UMAP dimensionality reduction. Cells belonging to the selected cell types were extracted and stratified into PTSD and control groups based on their sample identity (orig.ident). Each group-specific subset was converted into a cell_data_set (cds) object using Monocle 3’s new_cell_data_set() function. To ensure consistency in downstream analysis, the original Seurat UMAP embeddings and clustering labels were transferred into the Monocle object, avoiding the need for re-computation of dimensionality reduction or clustering.

Each cds object was preprocessed using preprocess_cds() with num_dim = 50 and norm method = “none”. The trajectory graph was learned using learn graph() without re-clustering. Cells were ordered along the trajectory using order_cells(), with the root node defined as the principal graph node most proximal to a designated reference population, typically the cell subtype presumed to represent an early or homeostatic state. Pseudotime values were visualized using plot_cells(), displaying cells colored by pseudotime and overlaid with the inferred trajectory graph. Control and PTSD groups were analyzed and visualized independently to highlight condition-specific transcriptional trajectories.

### 4.7 Ligand–receptor interaction analysis

Cell–cell communication networks were inferred using the CellChat (v2.1.2) package. Normalized expression data from Seurat objects were input into CellChat to estimate incoming and outgoing communication probabilities among neurovascular-related cell types. Communication probability matrices were calculated for both control and PTSD groups using the computeCommunProb and aggregateNet functions. Significant ligand–receptor pairs (p < 0.05) were visualized by heatmaps and network circle plots. Comparisons of interaction strength between groups were performed using the compareInteractions() function.

### 4.8 Gene ontology and pathway scoring

Module scores for biological processes such as Gobp_cellular_response_to_stress, Reactome _attenuation_phase, Go_inflammatory_response and Gobp_cytokine_mediated_signaling_pathway were calculated using Seurat’s AddModuleScore function based on curated GO biological process (GOBP) gene sets. Statistical significance between control and PTSD groups was assessed using two-tailed Wilcoxon tests.

### 4.9 Data visualization

All data visualizations including UMAP plots, violin plots, heatmaps, volcano plots, dot plots, and network diagrams were generated using ggplot2, ComplexHeatmap, and CellChat’s built-in functions in R (v4.4.1).

## Data Availability

All data generated or analyzed in this study have been deposited in the GEO repository under accession number GSE313257 and are publicly available at https://www.ncbi.nlm.nih.gov/geo/query/acc.cgi?acc=GSE313257.
