# Supplementary material for: Immune imbalance in the human hippocampus in PTSD revealed by single-nucleus transcriptomics
Source: Front Immunol. 2026 Jan 2;16:1697171. doi: 10.3389/fimmu.2025.1697171 (PMC12807954; doi:10.3389/fimmu.2025.1697171)
Supplement: Supplementary file 1 [file DataSheet1.pdf]

Immune imbalance in the human hippocampus in PTSD revealed by single-nucleus transcriptomics

Liu Liu[1], Pengfei Li[1], Brent A. Wilkerson[2], Yan Wu[3], Meng Liu[3], Roger Shi[1], Eric D. Hamlett[1], Steven L. Carroll[1], Amanda C. LaRue[1,4], Zhewu Wang[3,4]and Hongkuan Fan[1]*

*** Correspondence:**
Hongkuan Fan
fanhong@musc.edu

Figure S1: UMAP visualization and cell type proportions in control and PTSD samples

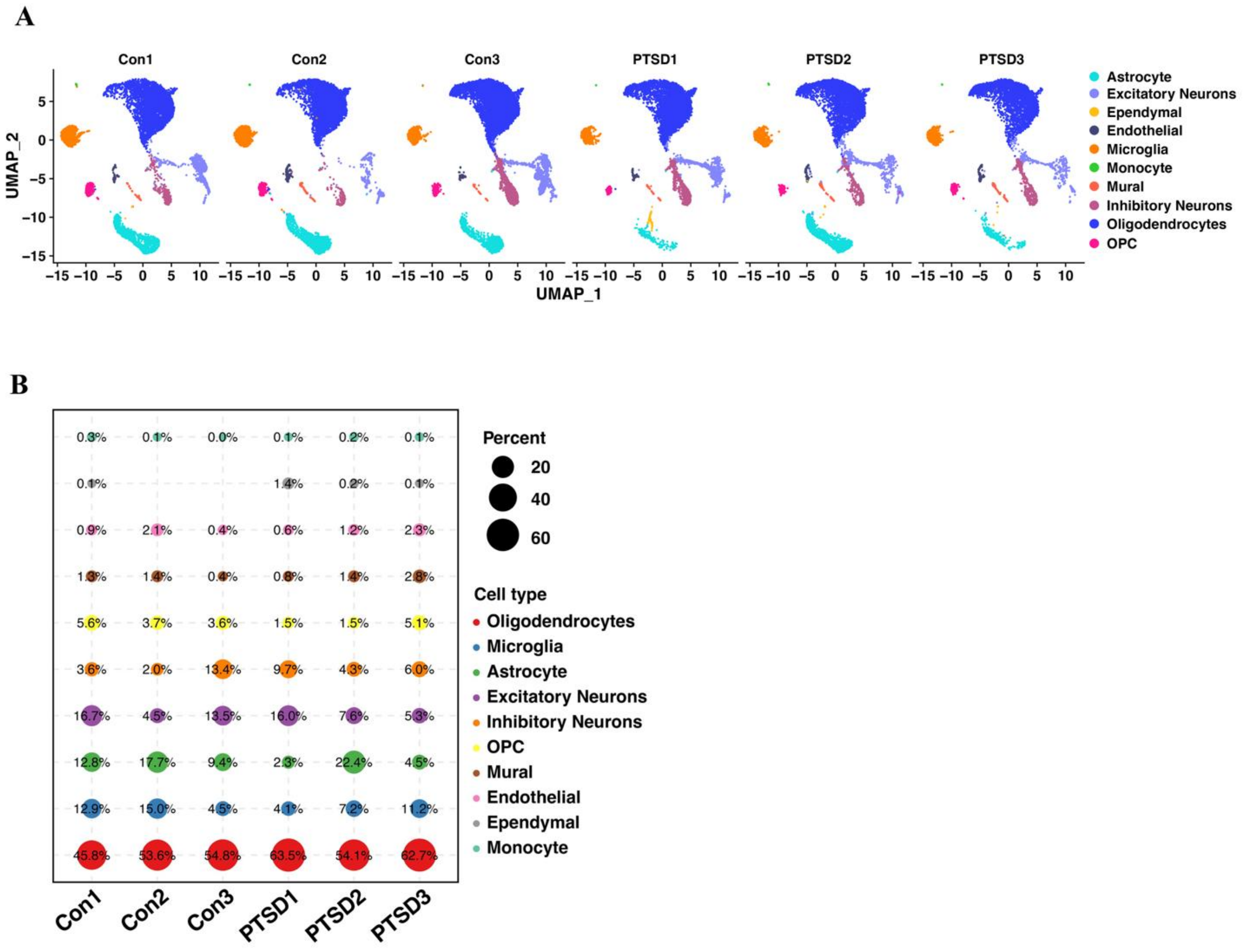

Figure S1. (A) Uniform Manifold Approximation and Projection (UMAP) plots illustrating the distribution of 10 transcriptionally distinct cell types in hippocampal tissues from three control and three PTSD donors. Each plot represents an individual donor, demonstrating consistent clustering across samples. **(B)** Bubble plots showing the relative proportions of the 10 identified cell types in hippocampal tissues from three control and three PTSD donors.

Figure S2: Differentially expressed genes in different cell types

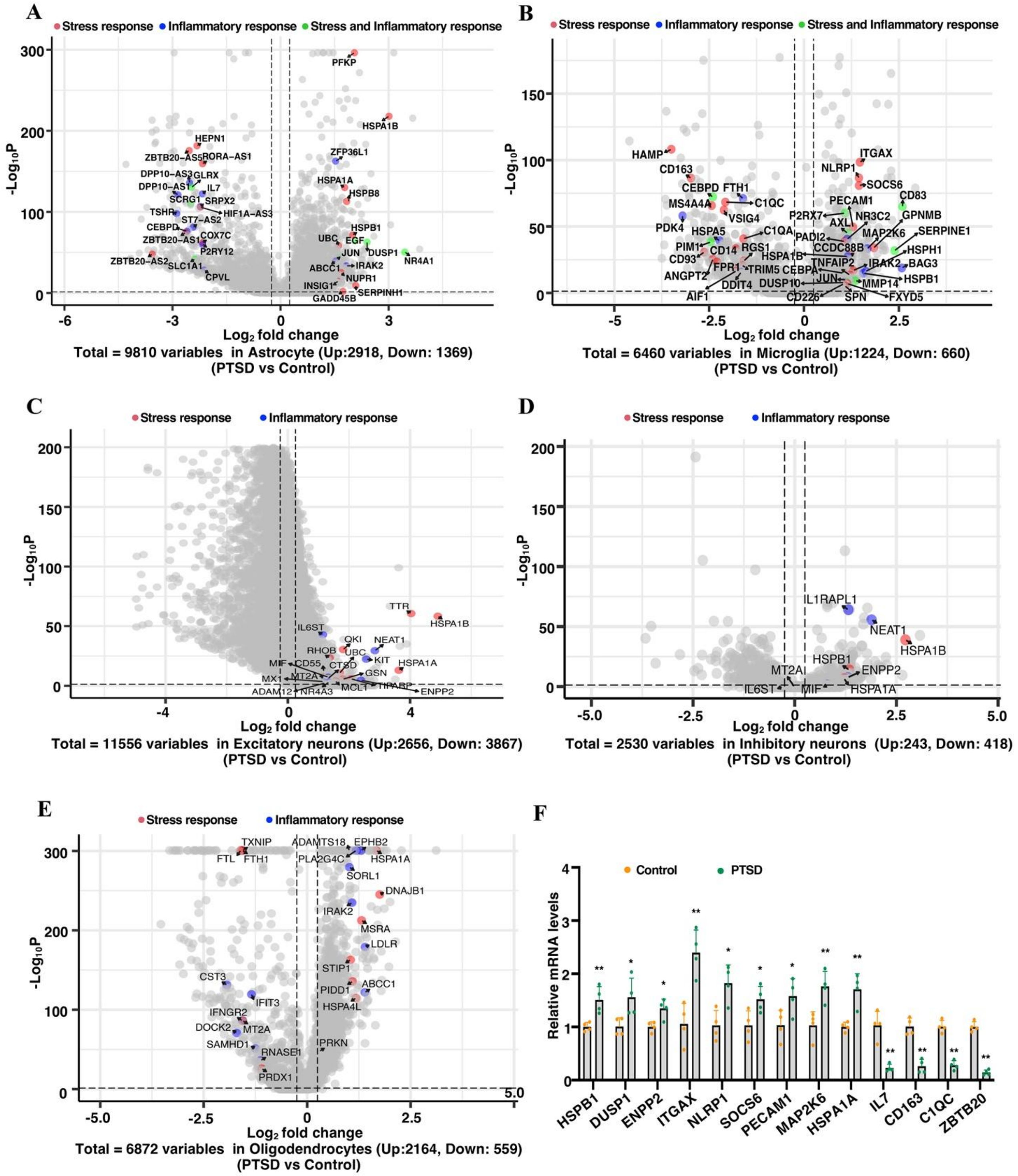

Figure S2. (A) Volcano plot of differentially expressed genes (DEGs) in astrocytes. Red, stress response–related genes; blue, inflammation-related genes; green, genes involved in both stress response and inflammation. (B) Volcano plot of differentially expressed genes (DEGs) in microglia. Red, stress response–related genes; blue, inflammation-related genes; green, genes involved in both stress response and inflammation. (C) Volcano plot of differentially expressed genes (DEGs) in excitatory neurons. Red, stress response–related genes; blue, inflammation-related genes. (D) Volcano plot of differentially expressed genes (DEGs) in inhibitory neurons. Red, stress response–related genes; blue, inflammation-related genes. (E) Volcano plot of differentially expressed genes (DEGs) in oligodendrocytes. Red, stress response–related genes; blue, inflammation-related genes. (F) Total RNA was extracted from the hippocampal tissue of Control and PTSD individuals, and the expression of representative DEGs was quantified by qPCR. Data are means ± SD ($n = 4$). *$P < 0.05$ and **$P < 0.01$, two-tailed t test.

Figure S3: Gene set enrichment analysis (GSEA) in excitatory and inhibitory neurons

A

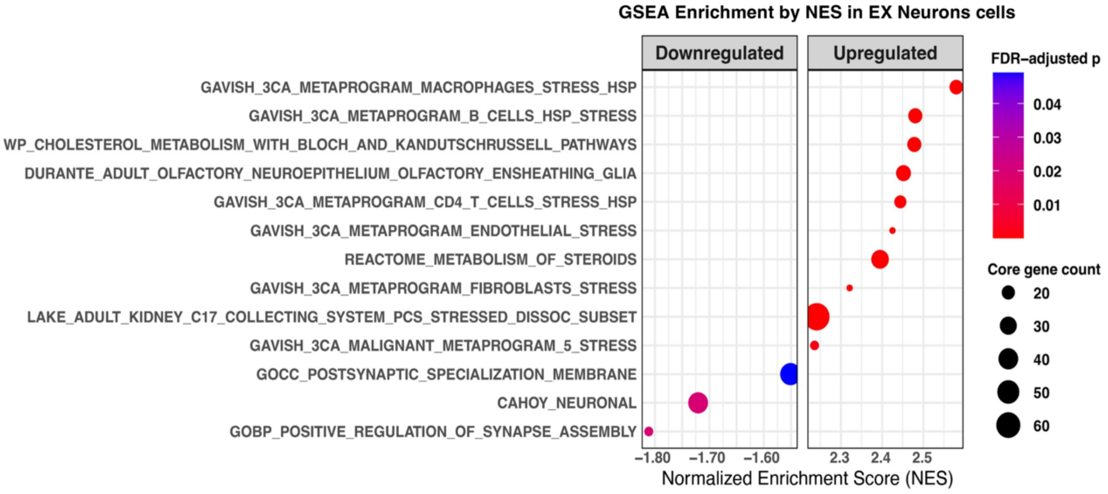

B

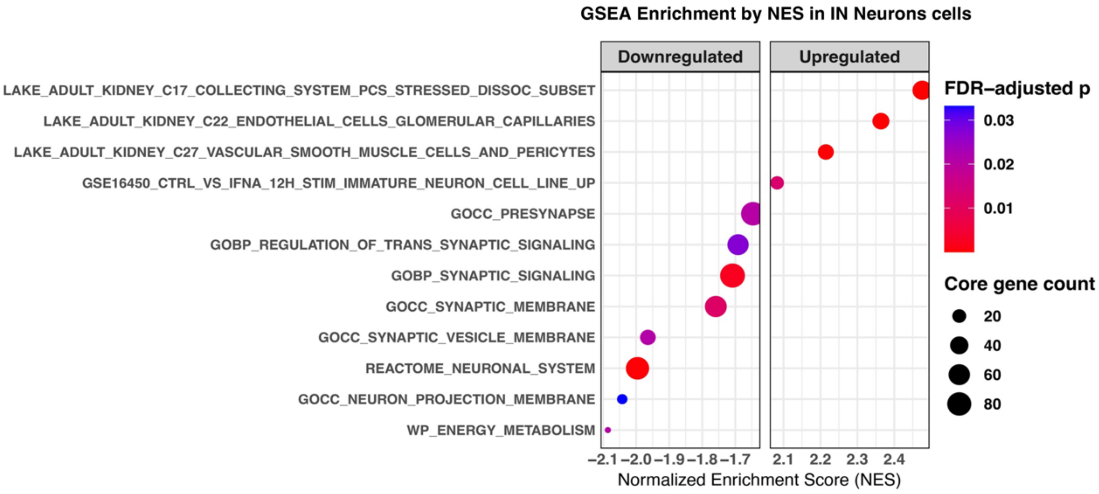

Figure S3. (A) GSEA results for excitatory neurons in PTSD versus control hippocampal tissues, showing significant enrichment of upregulated stress response gene sets (B) GSEA results for inhibitory neurons in PTSD versus control hippocampal tissues.

Figure S4: Gene set enrichment analysis (GSEA) in oligodendrocytes

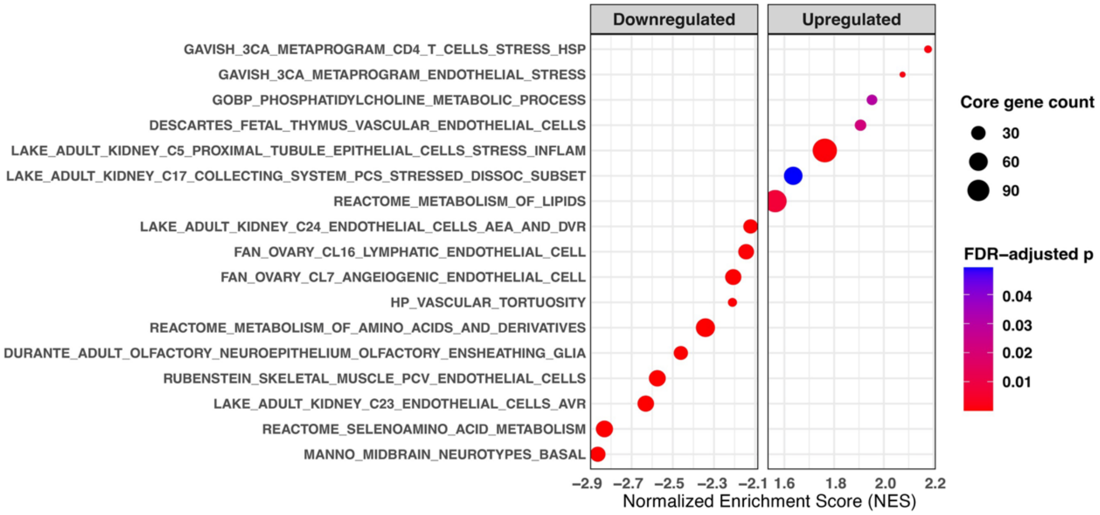

Figure S4. GSEA results for oligodendrocytes in PTSD versus control hippocampal tissues, revealing significant enrichment of upregulated stress response gene sets, indicating that stress-adaptive transcriptional programs extend to glial cells in the PTSD hippocampus.
